# Supplementary figures and images for: Metabolic profiling during COVID-19 infection in humans: Identification of potential biomarkers for occurrence, severity and outcomes using machine learning
Source: PLoS One. 2024 May 30;19(5):e0302977. doi: 10.1371/journal.pone.0302977 (PMC11139268; doi:10.1371/journal.pone.0302977)

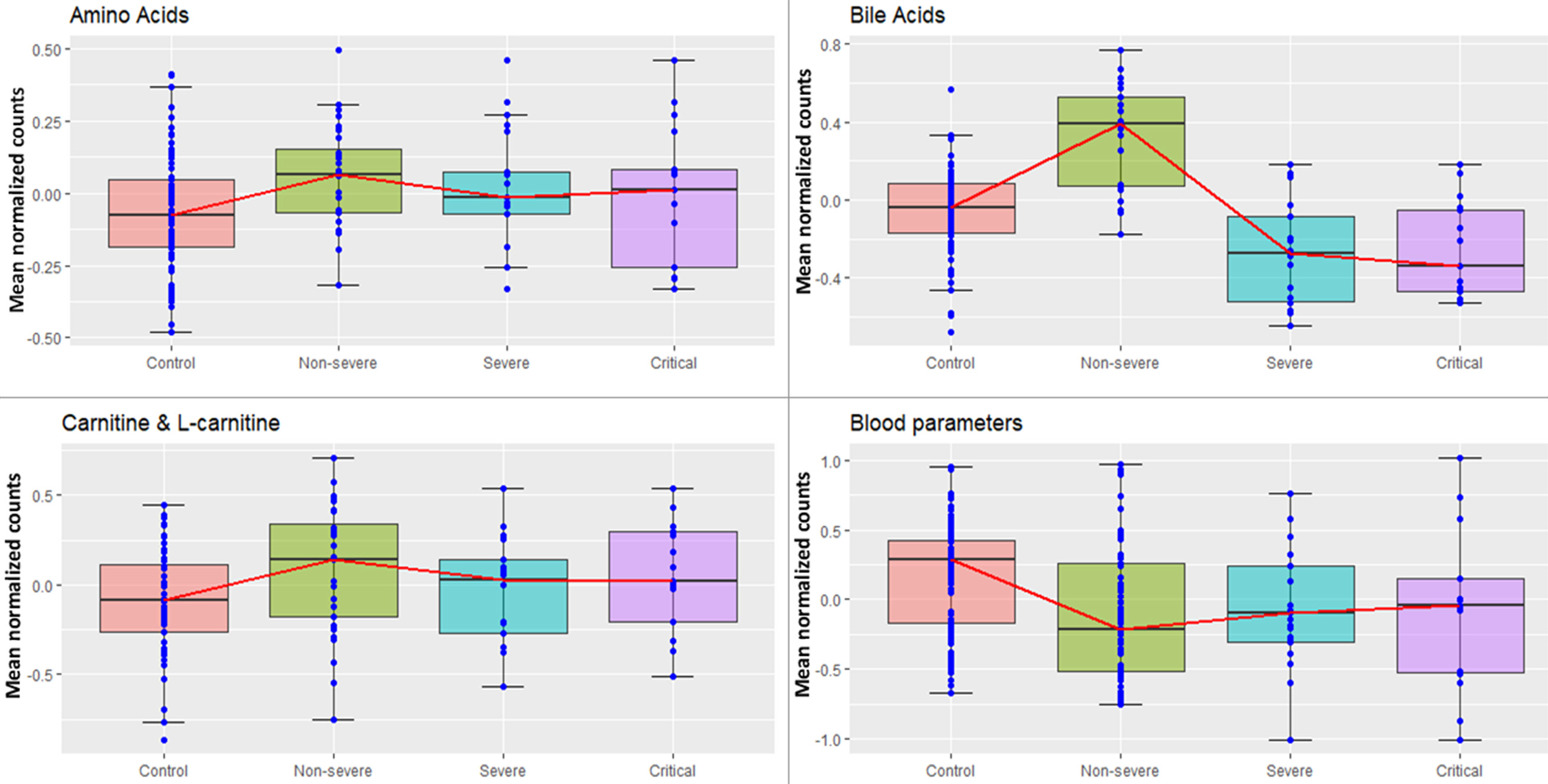

Supplement: S1 Fig — Each blue dot refers to mean normalized count of the respective metabolite subclass in one subject. The horizontal red line refers to the link between the median of the mean normalized counts across all groups and indicates the trends of change across multiple subject’s groups. Y. axes show the mean of normalized counts of metabolites in each subject. (TIF) [file pone.0302977.s001.tif]

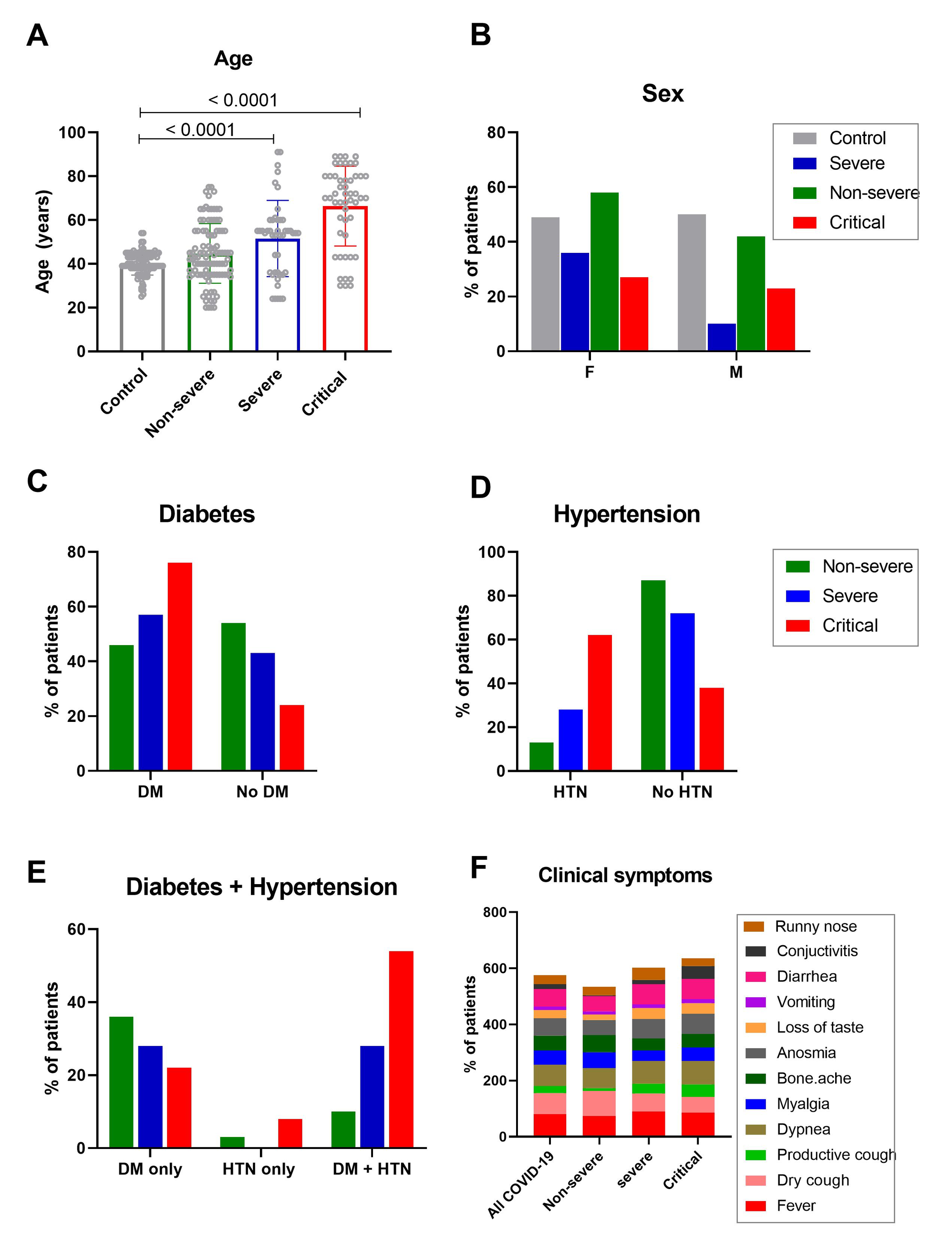

Supplement: S2 Fig — A. Age (years) of the study participants. Each dot refer to one patient. The significant differences among groups were calculated using one-way ANOVA with post-hock test at a cutoff P-value of 0.05. B. Distribution of sex in all participants. M: Male, F: Female. C-E. Proportions of COVID-19 patients that show respective comorbidity (diabetes, hypertension or both). HTN: Hypertension, DM: Diabetes. F. Proportion of COVID-19 patients showing different symptoms. (TIF) [file pone.0302977.s002.tif]

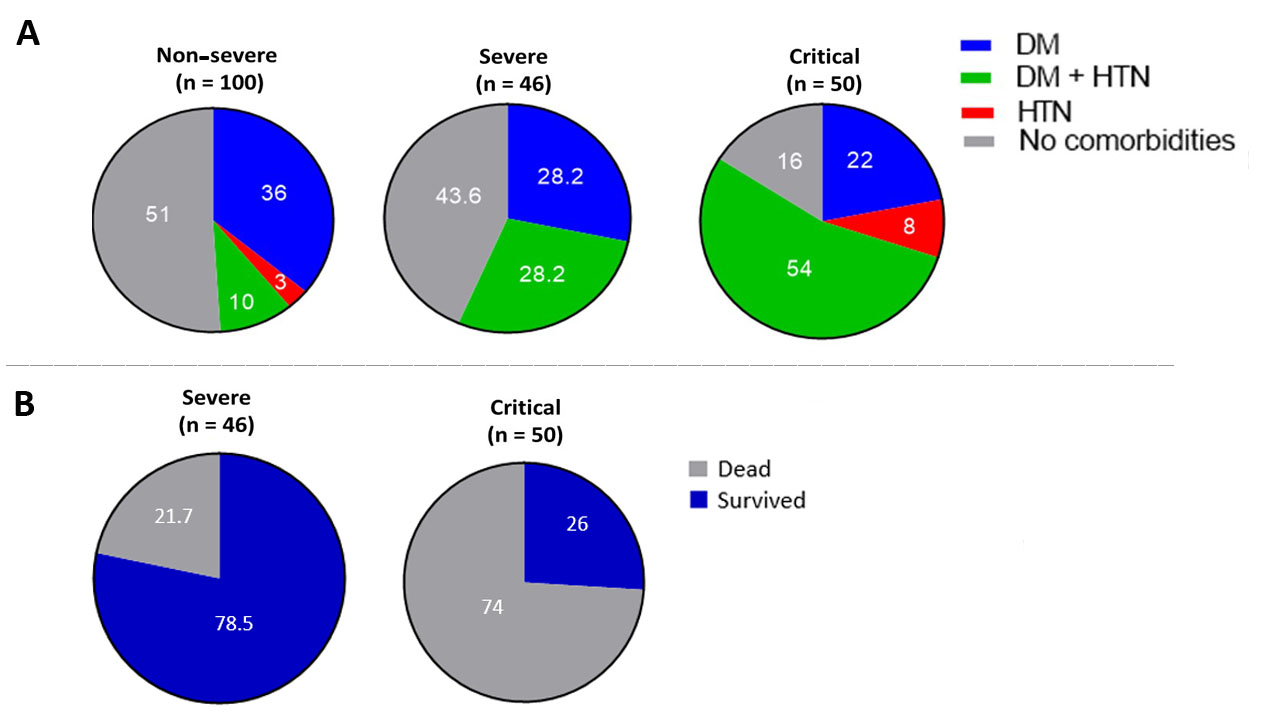

Supplement: S3 Fig — Frequency of occurrence of participants with and without comorbidities in patients group (A) and those with certain outcome (B). The figure shows proportions of respective class as a part of the total number of the patients within respective COVID-19 severity group. (TIF) [file pone.0302977.s003.tif]

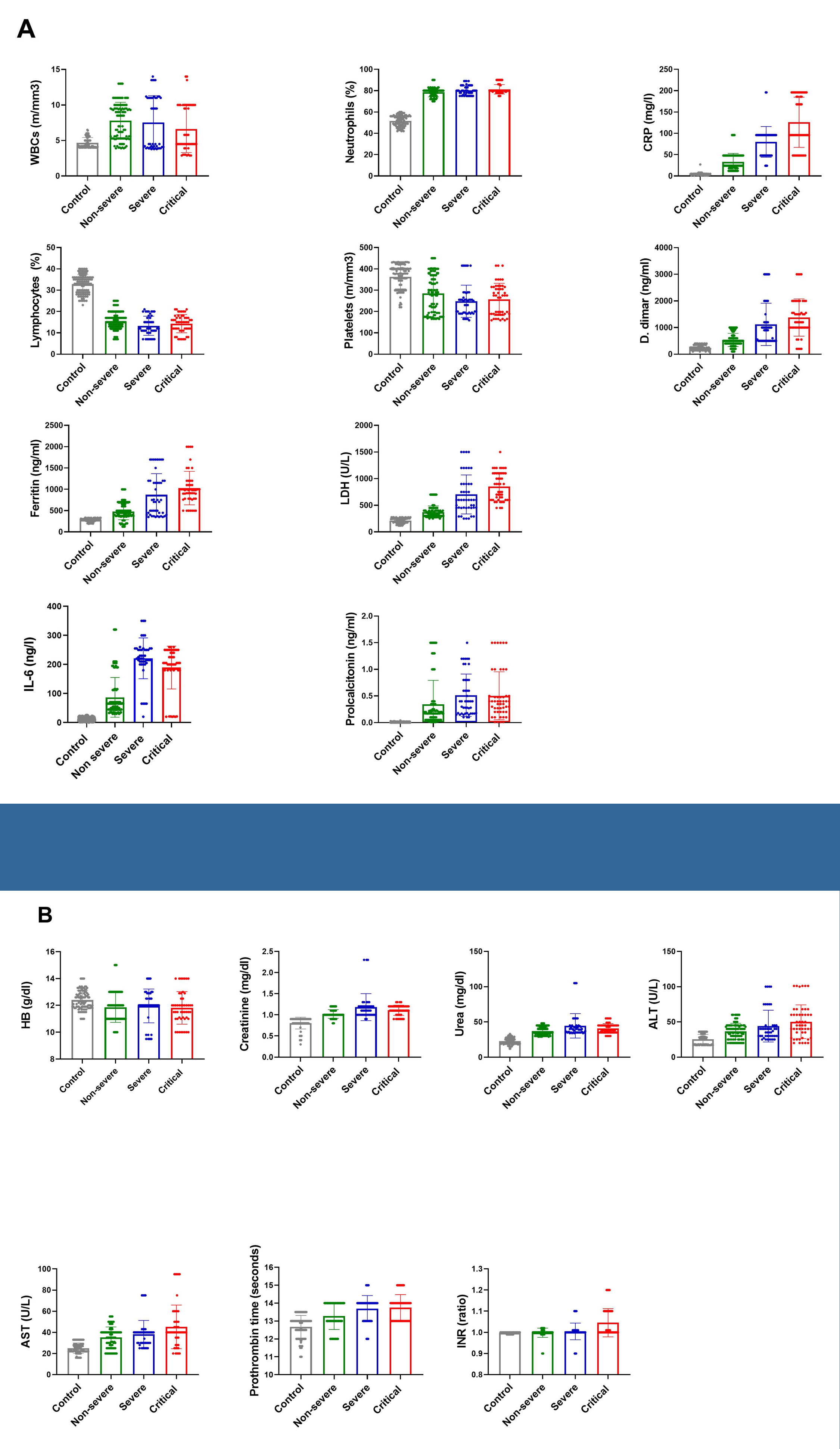

Supplement: S4 Fig — A & B. Values of blood parameters in controls and COVID-19 patients grouped by their disease severity. Each dot refers to one participant. Details of numerical values and statistical differences among groups for the laboratory parameters are shown in S2 Table. (TIF) [file pone.0302977.s004.tif]

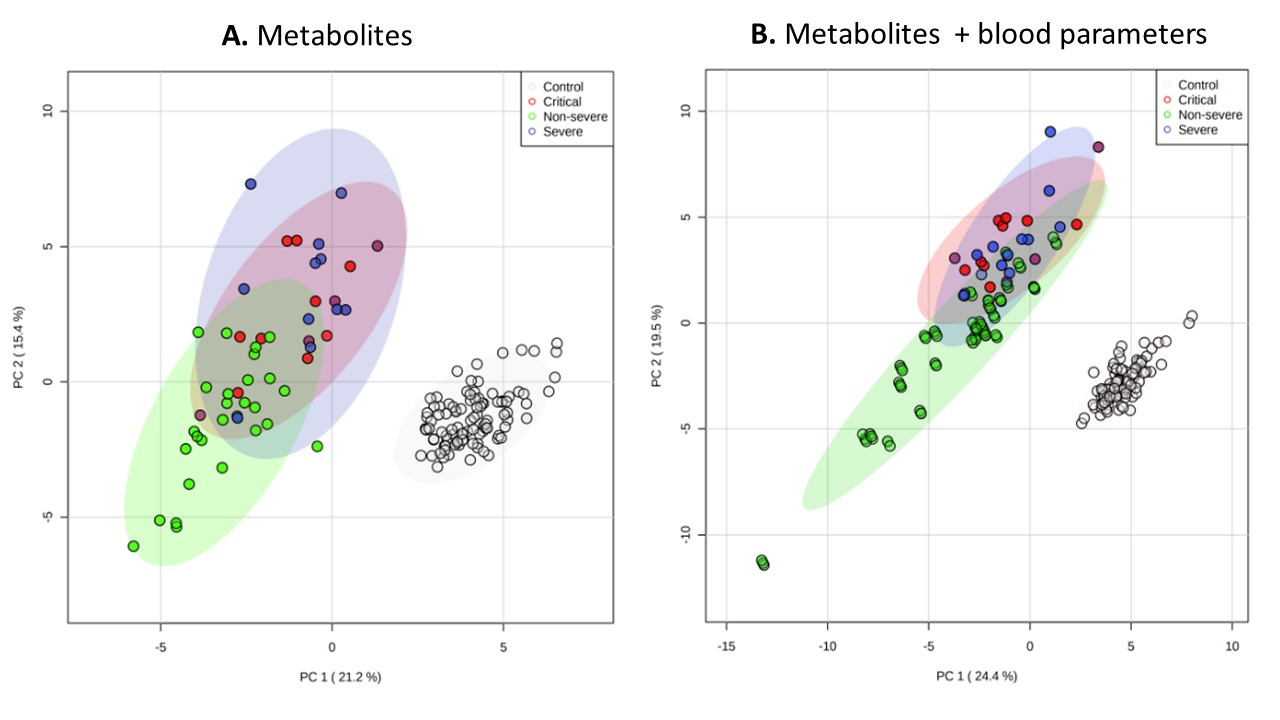

Supplement: S5 Fig — PCA plot of the study groups (shown as color-coded circles) based on the normalized counts of metabolites in all subjects (n = 295) (A) and metabolites + blood parameters concentration (B). The distance between points are the Euclidean distance. (TIF) [file pone.0302977.s005.tif]

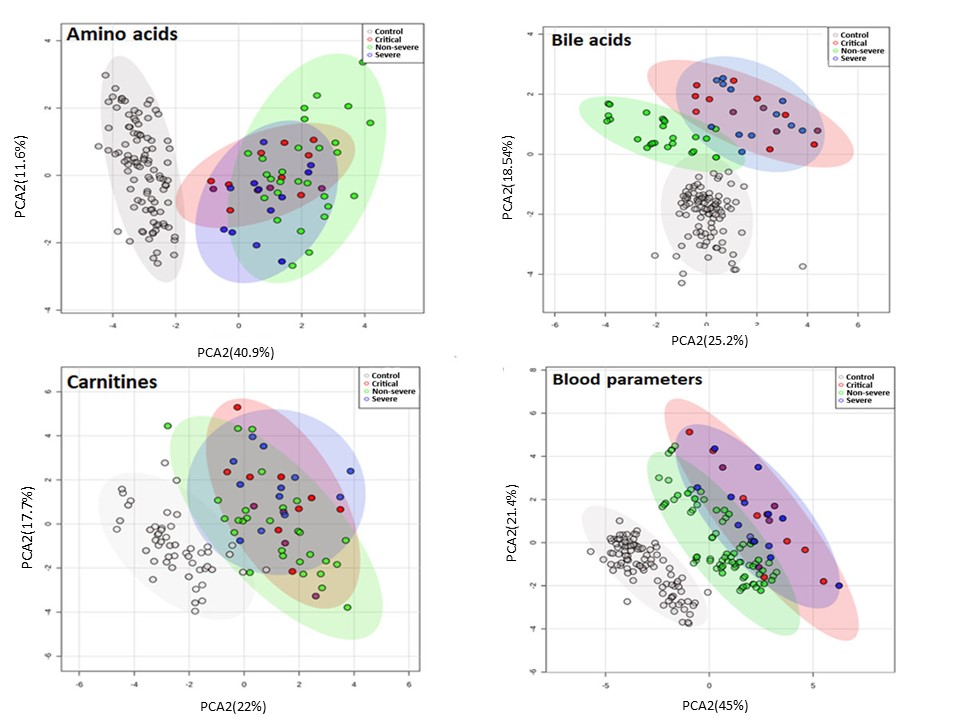

Supplement: S6 Fig — The distance between points are the Euclidean distance. (TIF) [file pone.0302977.s006.tif]

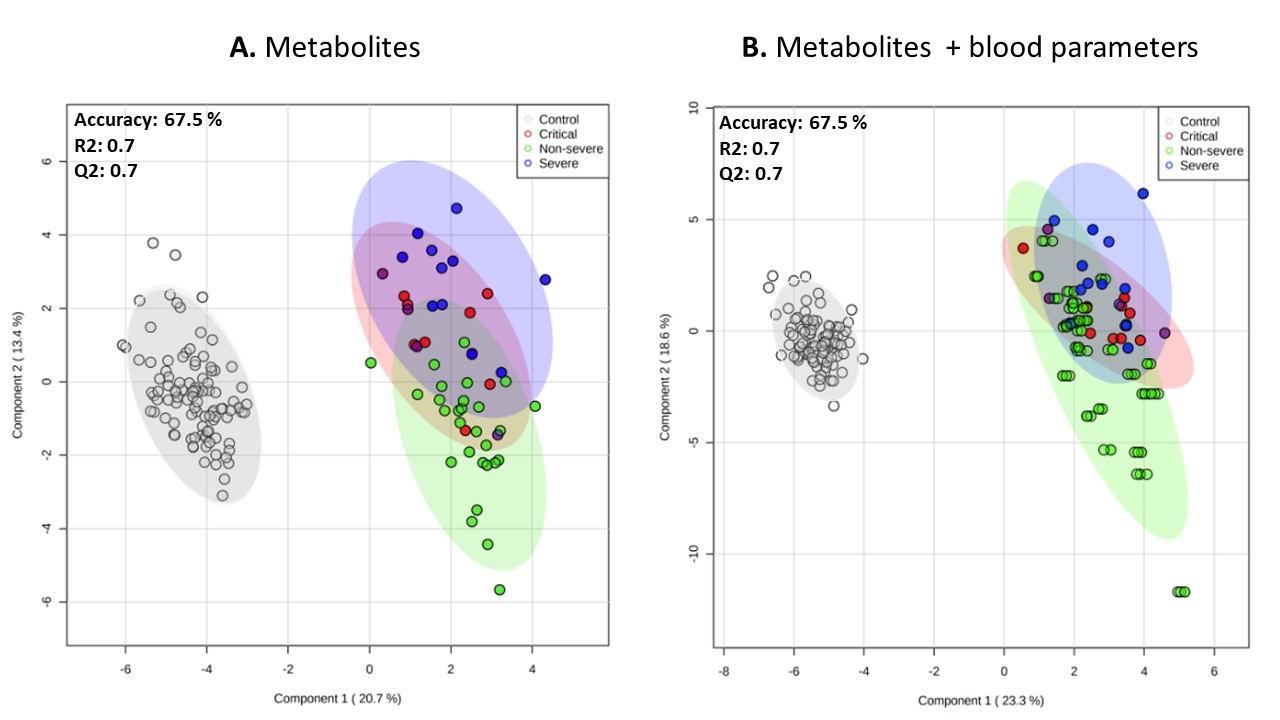

Supplement: S7 Fig — Score scatter plot of PLS-DA model showing the classification of patients in all groups based on the normalized concentration of all metabolites only (n = 50) (A) and based on normalized concentration of both metabolites and blood (B). Parameters for model evaluation are shown as accuracy, variation between classes (R2Y) and predictive ability (Q2Y). (TIF) [file pone.0302977.s007.tif]

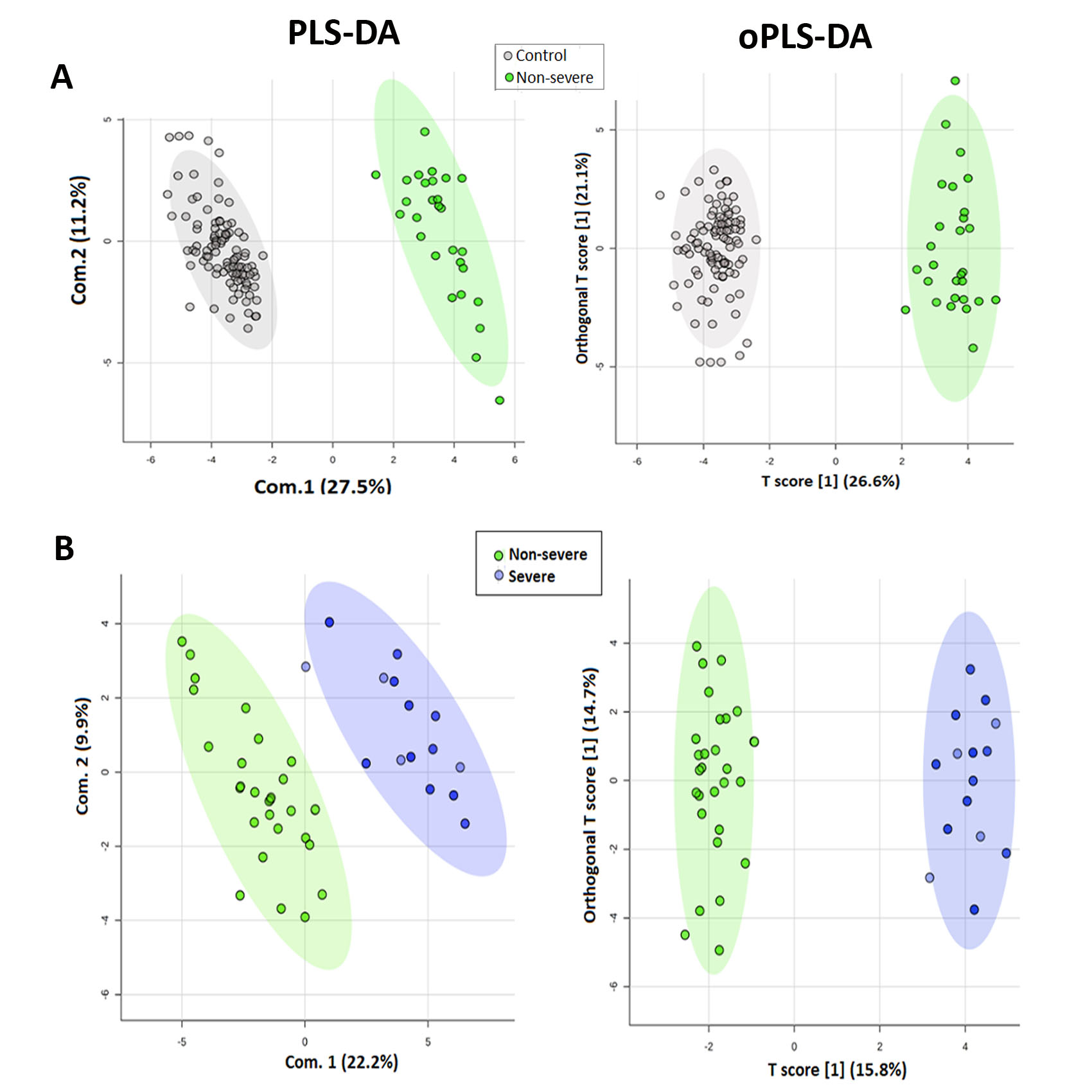

Supplement: S8 Fig — Score scatter plot of PLS-DA and oPLS-DA models comparing HC vs non-severe subjects (A) and non-severe vs severe patients (B). Each dot refers to one patient. (TIF) [file pone.0302977.s008.tif]

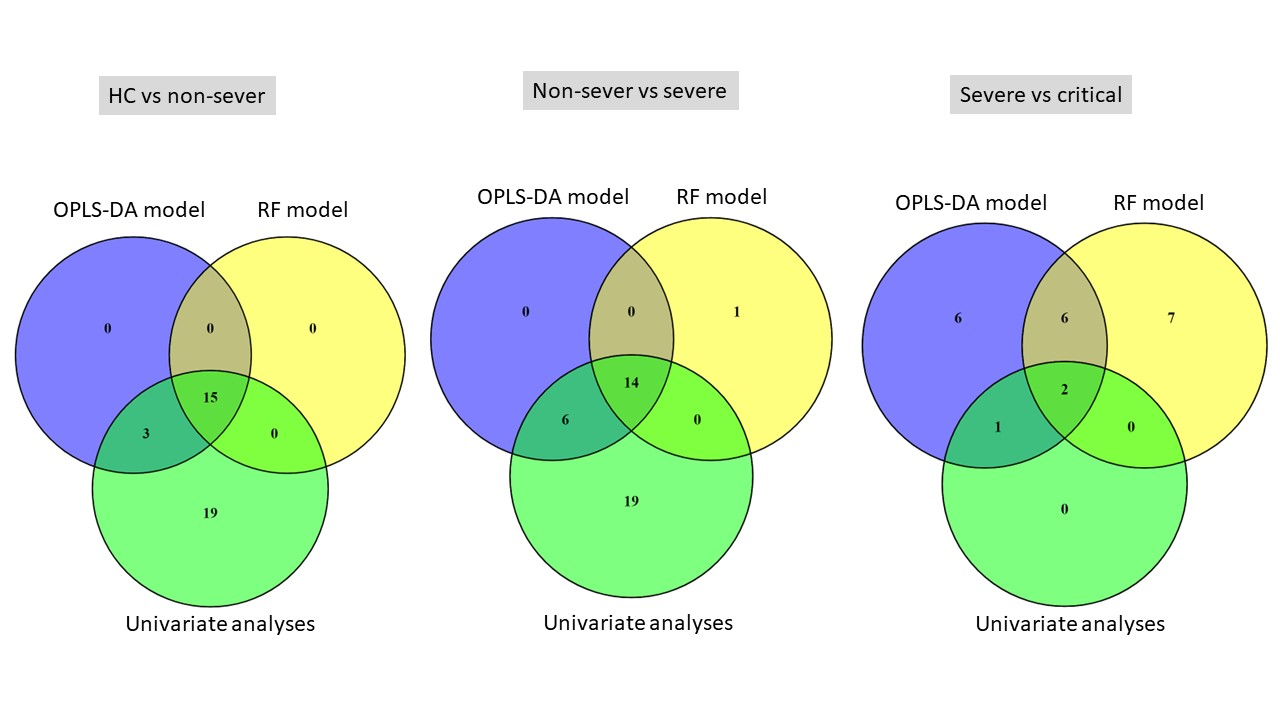

Supplement: S9 Fig — The middle intersection among the 3-approaches refers to panel 1, panel 2 and panel 3, details of which are shown in Table 3. (TIF) [file pone.0302977.s009.tif]

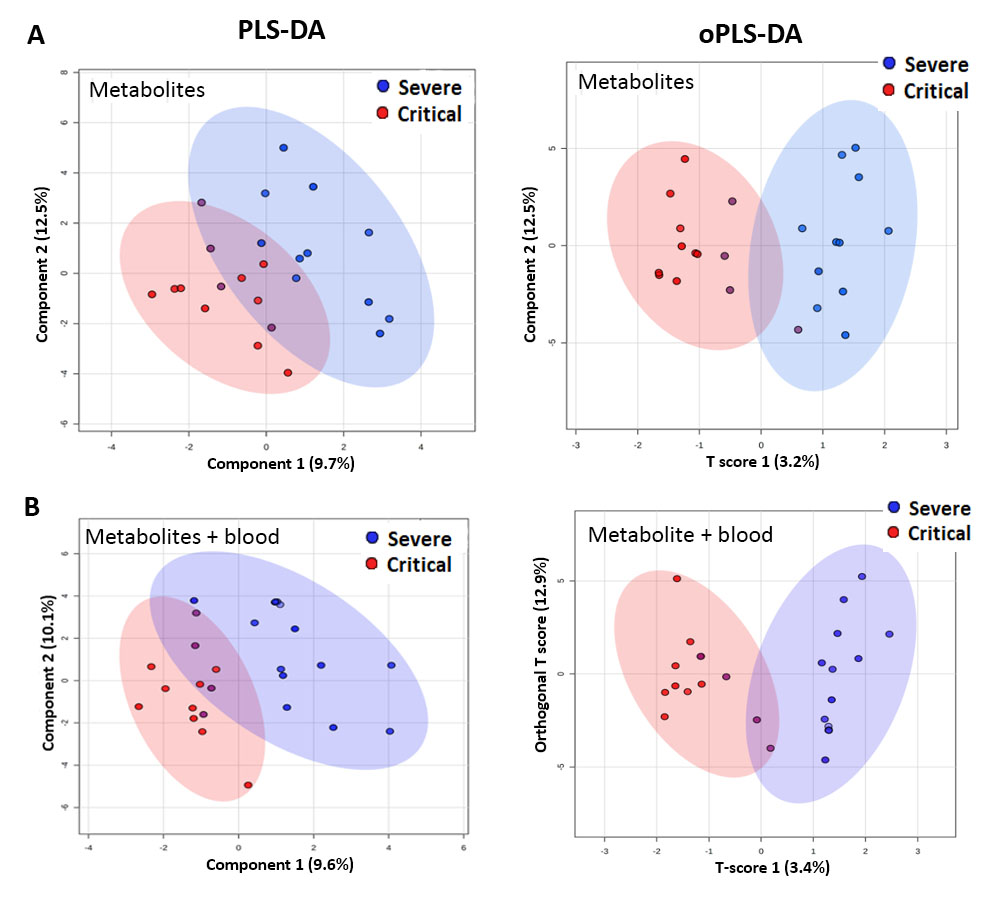

Supplement: S10 Fig — Score scatter plot of PLS-DA and oPLS-DA models discriminating severe and critical COVID-19 cases based on normalized concentrations of metabolites (A) and both metabolites and blood indices (B). Each dot refers to one patient. (TIF) [file pone.0302977.s010.tif]

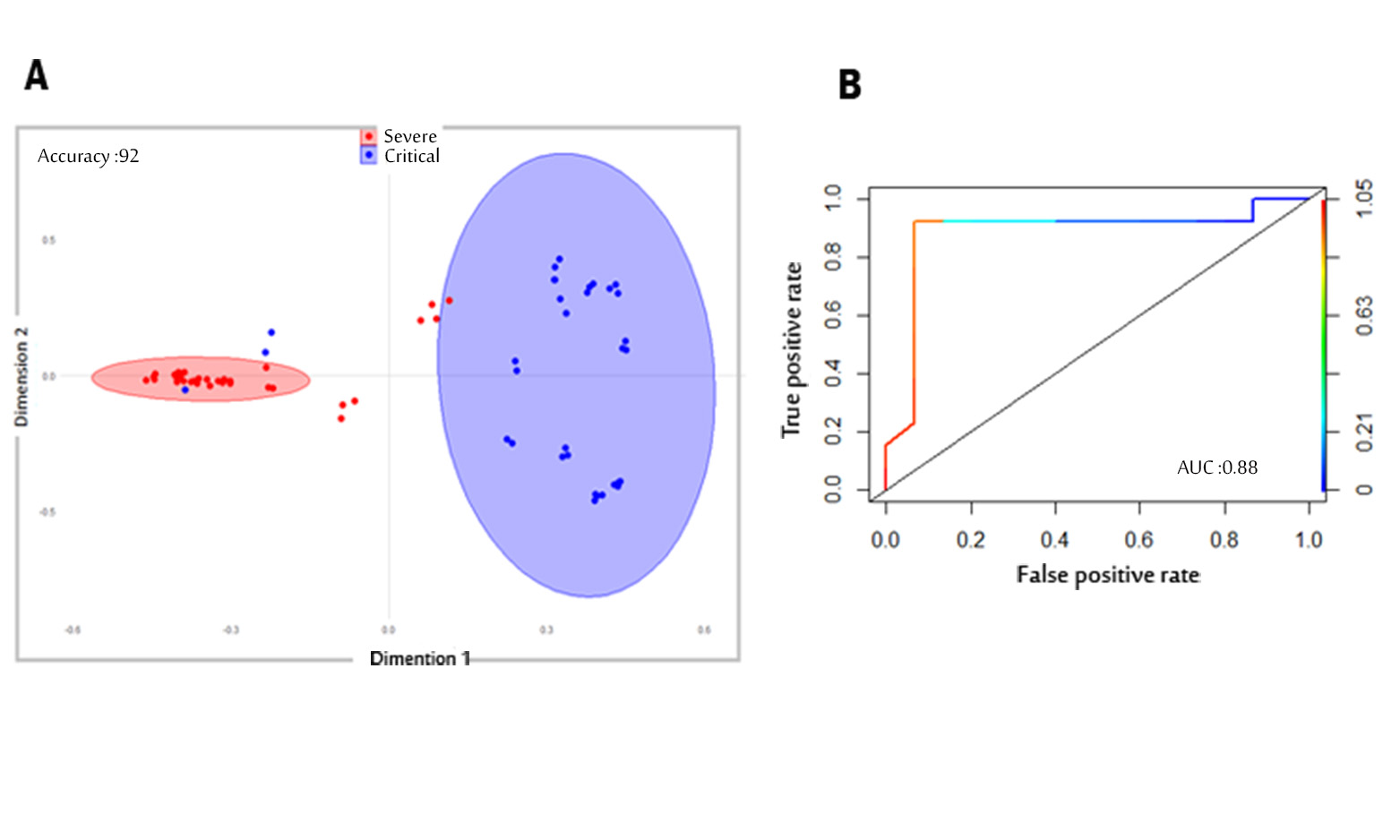

Supplement: S11 Fig — A. Score scatter plot showing the classification of both severe and critical. B. ROC analyses showing the predictability of the model as a classifier. AUC: Area under the curve. (TIF) [file pone.0302977.s011.tif]

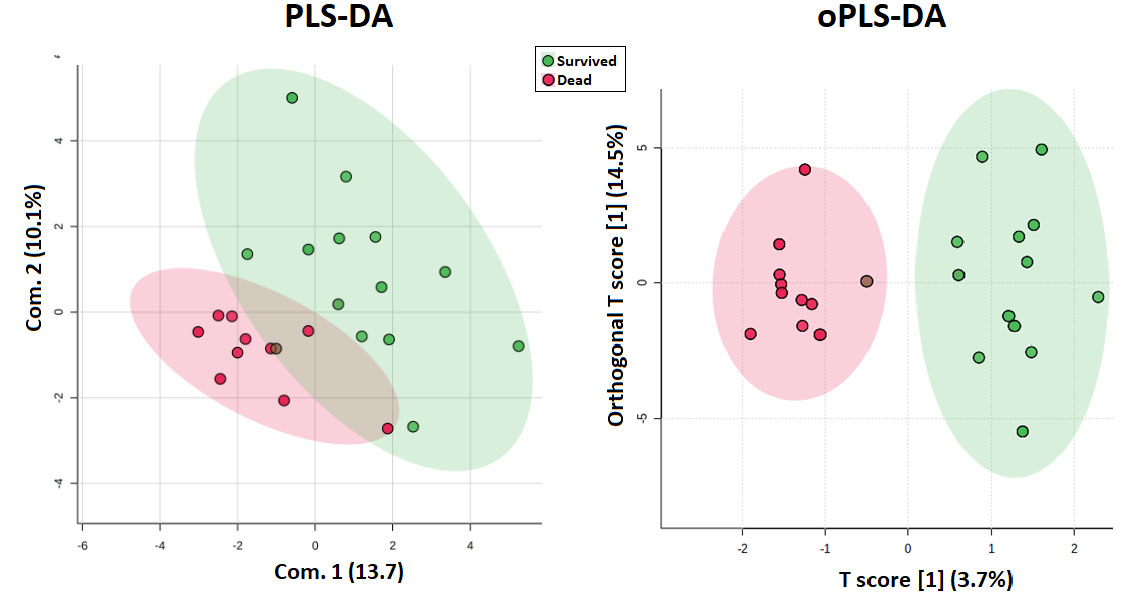

Supplement: S12 Fig — Score scatter plots showing the PLS-DA (A) and oPLS-DA models (B) using the normalized concentration of metabolites to classify patient’s outcomes (survived and dead). Each dot refers to one patient. (TIF) [file pone.0302977.s012.tif]

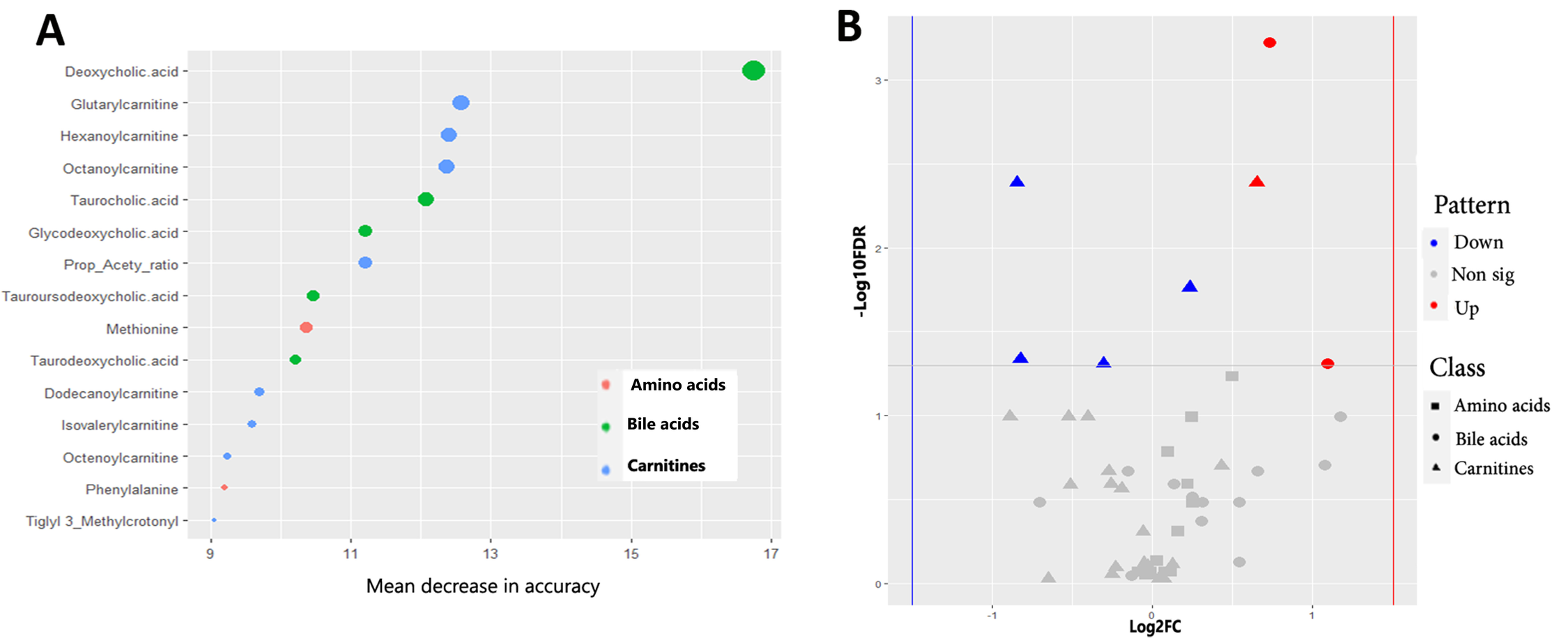

Supplement: S13 Fig — A. Top 15 metabolites that are important predictors for patient outcome as revealed by RF model. The metabolites are color-grouped by their class and are ranked descending by their mean decrease in accuracy (the higher the mean decrease in accuracy the more important the metabolite). B. Volcano plot showing the results of the univariate analyses. The figure depicts the relationship between log2FC value of each metabolite (x-axis) against its -log10FDR (y-axis). The pattern of differential expression of each metabolite are color-coded and their class are shape coded. The dashed horizontal line refers to 1.3, the–log10 for a 0.05 FDR. The vertical dashed lines refer to the cutoff that equates to a fold change value of |1.5|. (TIF) [file pone.0302977.s013.tif]

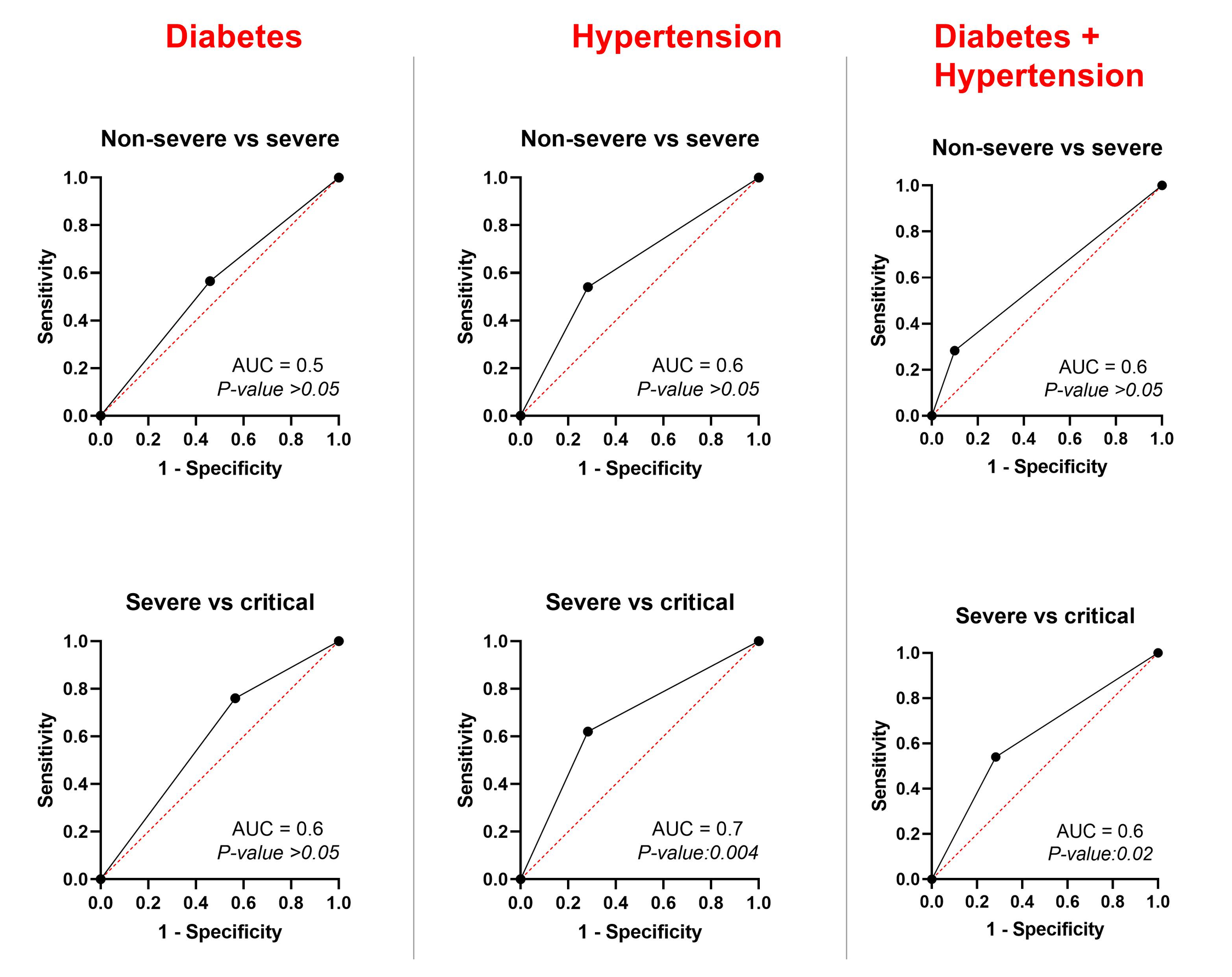

Supplement: S14 Fig — AUC: Area under the curve. (TIF) [file pone.0302977.s014.tif]
