# Supplementary material for: Metabolic profiling during COVID-19 infection in humans: Identification of potential biomarkers for occurrence, severity and outcomes using machine learning
Source: PLoS One. 2024 May 30;19(5):e0302977. doi: 10.1371/journal.pone.0302977 (PMC11139268; doi:10.1371/journal.pone.0302977)
Supplement: Attachment — Submitted filename: Metabolic profiling during COVID_Review comments.docx [file pone.0302977.s021.docx]

“Metabolic profiling during COVID-19 infection in humans: Identification of potential biomarkers for occurrence, severity and outcomes” by Elabd and co-workers studied the metabolic profiles of different groups of patients having different severity of covid 19. I recommend the editor to accept this publication after major revision on addressing the following queries.

1. As per Methods details blood sample are collected from covid patient between the year 2021 to 2022. When they analyze these samples using LC-MS in 2023 or between 2021 to 2022 immediately collecting after the blood sample collection?

If data is not collected immediately or after 1-2 month after the sample collection. How authors will justify the change in metabolites is due to severity of the covid 19 patient not due to ageing of the sample stored in refrigeration at -80°C?? Did the authors perform any control experiment to confirm any of the metabolites they found is due to sample aging or not??

2. In Table 1, authors mention % in front of symptom (like cough/fever etc.) each group. Does this % means, among the total number of patients in each group that percent of patients are with symptoms of cough, fever etc???
