# Supplementary material for: Metabolic profiling during COVID-19 infection in humans: Identification of potential biomarkers for occurrence, severity and outcomes using machine learning
Source: PLoS One. 2024 May 30;19(5):e0302977. doi: 10.1371/journal.pone.0302977 (PMC11139268; doi:10.1371/journal.pone.0302977)
Supplement: Attachment — Submitted filename: Comments.docx [file pone.0302977.s022.docx]

1. Section 2.2-Sample Collection:

Please specify the type/kind of vacutainer used, in text.

2. Section 4- Discussion:

Page 2, Paragraph 1.. "Antibiotic administered..... acid [54]."

Please review the statement for accuracy and rephrase

if required. Does the infection cause the reduction

in gut microbiome diversity or the antibiotics?

Further, please change nsp15 endonuclease to nsp15 endoribonuclease.

3. The manuscript requires an overall editing in

terms of consistency and grammar for tenses. Also for

sub/super-scripts.
