# Supplementary material for: Metabolic profiling during COVID-19 infection in humans: Identification of potential biomarkers for occurrence, severity and outcomes using machine learning
Source: PLoS One. 2024 May 30;19(5):e0302977. doi: 10.1371/journal.pone.0302977 (PMC11139268; doi:10.1371/journal.pone.0302977)
Supplement: Attachment — Submitted filename: Response to Reviewers.docx [file pone.0302977.s023.docx]

Dear Anil Bhatia,

We would like to sincerely thank you and the external reviewers for the full consideration of our submitted manuscript and the valuable comments that will help us improving the quality of our manuscript entitled “Metabolic profiling during COVID-19 infection in humans: Identification of potential biomarkers for occurrence, severity and outcomes”, PONE-D-23-40352.

In the revised version of the manuscript (file name: “revised manuscript with track changes”), we have implemented the required comments given by the academic editor and the reviewers. Please find below the point-by-point rebuttal for the comments. We hope that the new version is qualified**.**

- We have added the words “using machine learning” at the end of the title. Indeed, this addition will make the title more accurately reflecting the core work that have been done in this manuscript. The whole work is centered on using 3-machine learning models to predict and identify potential metabolites that are diagnostic and prognostic biomarkers. These models were further used in other parts of the manuscript. The work described in this manuscript represents a vivid example where biomedical data is processed by machine learning approaches to reach clinically relevant insights. Therefore, it was important to add this in the title
- **Journal requirements**

1. Please ensure that your manuscript meets PLOS ONE's style requirements, including those for file naming. The PLOS ONE style templates can be found at
   https://journals.plos.org/plosone/s/file?id=wjVg/PLOSOne_formatting_sample_main_body.pdf and <https://journals.plos.org/plosone/s/file?id=ba62/PLOSOne_formatting_sample_title_authors_affiliations.pdf>

The manuscript has been formatted according to the PLOS ONE’s style requirements.

2. Please ensure that you have specified a) Did participants provide their written or verbal informed consent to participate in this study? b) If consent was verbal, please explain i) why written consent was not obtained, ii) how you documented participant consent, and iii) whether the ethics committees/IRB approved this consent procedure.

Under the section “consent to participants”, we have clarified that the patients were offered to sign an informed written consent prior to sample collection

3. The name of the colleague or the details of the professional service that edited your manuscript

After implementing the reviewer’s comments, the manuscript has gone throughout a 2^nd^ revision for words and grammars, including tenses, by the two corresponding authors of the manuscript (Dr. Mohamed Samir, lecturer in Bath University, UK and Dr. Naglaa S. Elabden, assistant professor of tropical medicine, Faculty of Medicine, Menoufia University, Egypt), and then the revised version was thoroughly checked by all authors. Please see some edits in the edited version of the manuscript (file name: ”revised manuscript with track changes”).

A copy of your manuscript showing your changes by either highlighting them or using track changes (uploaded as a *supporting information* file)

We have provided this copy under file name: “revised manuscript with track changes”

A clean copy of the edited manuscript (uploaded as the new *manuscript* file)”

We have provided this copy under file name “manuscript”

4. Thank you for stating the following financial disclosure: "The UPLC MS/MS instrument was a grant from Science and Technology Development Fund (STDF), Egypt. Grant number: N2338."

This funding enabled the establishing of the UPLC MS/MS instrument in our laboratory. We have used this instrument to measure the metabolite concentrations in this study.

5. We note that you have included the phrase “data not shown” in your manuscript. Unfortunately, this does not meet our data sharing requirements. PLOS does not permit references to inaccessible data. We require that authors provide all relevant data within the paper, Supporting Information files, or in an acceptable, public repository. Please add a citation to support this phrase or upload the data that corresponds with these findings to a stable repository (such as Figshare or Dryad) and provide and URLs, DOIs, or accession numbers that may be used to access these data. Or, if the data are not a core part of the research being presented in your study, we ask that you remove the phrase that refers to these data.

Thanks for your comment. We have removed the phrase “data not shown” because we found that these data are not a core part of the research.

6. Your ethics statement should only appear in the Methods section of your manuscript. If your ethics statement is written in any section besides the Methods, please move it to the Methods section and delete it from any other section. Please ensure that your ethics statement is included in your manuscript, as the ethics statement entered into the online submission form will not be published alongside your manuscript.

Thanks for your comment. We have moved the ethical statement to the method section.

- **Reviewer’s comments**

**Reviewer #1:** “Metabolic profiling during COVID-19 infection in humans: Identification of potential biomarkers for occurrence, severity and outcomes” by Elabd and co-workers studied the metabolic profiles of different groups of patients having different severity of covid 19. I recommend the editor to accept this publication after major revision on addressing the following queries.

Many thanks for your recommendations to make our work more valuable and scientific enough to be published in the PLOS One journal. We took all the points you mentioned in our considerations and tried to enhance our work by adding your modifications and recommendations for each point.

1- As per Methods details blood sample are collected from covid patient between the year 2021 to 2022. When they analyze these samples using LC-MS in 2023 or between 2021 to 2022 immediately collecting after the blood sample collection?
   If data is not collected immediately or after 1-2 month after the sample collection. How authors will justify the change in metabolites is due to severity of the covid 19 patient not due to ageing of the sample stored in refrigeration at -80°C?? Did the authors perform any control experiment to confirm any of the metabolites they found is due to sample aging or not??

Thanks for your comment. We can ensure that sample aging is not biasing our metabolite measurements because we have analyzed the samples for metabolites with no delay. Briefly and as we mentioned in the material and method section, the filter papers that were used for carnitine and amino acids measurements were stored at -80 and were analyzed within 1-month of collection, which is not affecting the measurements of these metabolites according to the guidelines in the used kit (MassChrom® Amino Acids and Acylcarnitines from Dried Blood / Non Derivatised-LC-MS/MS, Order No.: 57000/F). Furthermore, serum bile acids were also analyzed within 1-month of the sample collection. Indeed, we have previously confirmed that bile acid concentrations in not affected by storage in -80 nor by few freezing/thawing cycles [Khalil et al. 2021, https://doi.org/10.1186/s43066-021-00142-3).

2- In Table 1, authors mention % in front of symptom (like cough/fever etc.) each group. Does this % means, among the total number of patients in each group that percent of patients are with symptoms of cough, fever etc???

Thanks for your comment. The percentages mentioned in table 1 (including in the symptoms) represent the proportion of subjects having particular gender, score, or symptoms out of the total number of subjects in the respective COVID-19 group. For clarity, we have indicated this as a footnote under table 1.

**Reviewer #2:** The review explains all the possible metabolites from blood samples which might be helpful in prediction of the stage of COVID -19 patients and severity of the disease. I have some concerns listed below:

Many thanks for your valuable comments and we are sure that your recommendations will enforce our work. So, we tried to reply to each point and modified our manuscript according to your recommendations.

1. When the authors categories the various stages of COVID-19 patients (section 2.1), they wrote that severity is considered with any of the following include low oxygen saturation level which they defined below 90 and respiratory distress whereas Critical Patients who need the mechanical ventilation. My concern is that some patients might have low oxygen saturation and does use all CPAP and other oxygen flow ways. During that stage, some patients need more oxygen flow from outside and some might need less depending upon the lungs functioning. How can we compare when the patients are in early stages of virus and need less outside oxygen flow (may be 4 or 6) versus patients in late stages of disease and need more oxygen flow (may be 14). The latter patients might also be similar to critical ones.

Thanks for this valuable comment. It is well known that severely affected COVID-19 patients are frequently presented with a wide spectrum and the overlapped symptomatology. With the scope of this study trying to reveal stage-specific biomarkers and this varied symptomatology, we sampled the patients at their admission and then classified them relying on the published WHO guidelines, in which severe patients were determined as having oxygen saturation less than 90% irrespective of their severity stage. We do however agree with your opinion that later in the course of the illness some of those severe patients needed oxygen therapy that was provided via nasal cannula or mask according to the lung condition, but this does not affect our metabolite measurements as we took the samples earlier. Furthermore, the critical patients included those who were classified and sampled at their admission.

Secondly, sometime during the ventilation, there is ventilated pneumonia associated with disease, how that can be ruled out.

Thanks for your comment. For severe and critical patients, all samples were taken from the patients right at the admission and before being set on a ventilator. This is a routine procedure at our diagnostic facilities. So, there is no possibility of ventilation-associated pneumonia. We have added this in the respective section of the edited version of the manuscript.

2. What age group is used during data collection? As per I understand, Is the age graph is one which represented in supporting figure S2, if that is true, how can we compare metabolites samples from age group 20-30 years versus age group 70-80 years? Please comment on that.

Thanks for your valuable comment. You are true in that subject’s ages are visualized in Figure S2 (as we mentioned in the result section). Indeed, it was hard to select matched age groups knowing that old age is a well-known risk factor for critical and sever COVID-19 infection. However, our previous research on metabolites in other viral disease contexts (e.g. hepatitis C virus infection) confirmed that patient age is not confounding the measured metabolites. In these research, for measuring metabolites, we have been routinely using single reference for all adult groups who are > 18-years old with no further categorization of the adult group and in the current study, none of our subjects lie below 20 years old.

3. During patient treatment on severity and critical stages of disease, there are many medications given to patients. How can we rule out some metabolites might alter in the blood because of that medication. Please comment.

Indeed, non-severe patients who participated in the study at that stage and according to the protocol were found to have taken antipyretics and analgesics that are known to have no effect on host metabolite levels. For severe and critically ill patients, the samples used for this study were taken prior to the start of any medication (i.e. at the first admission or diagnosis). However, for some of the patients, who were already admitted at the critical phase, we do not have information about their previous medication regime, which could have affected some of our measured metabolites. We have indicated this information in the respective sections in the edited version of the manuscript.

**Reviewer #3:** Manuscript titled as "Metabolic profiling during COVID-19 infection in humans: Identification of potential biomarkers for occurrence, severity and outcomes" I would like to congratulate the authors for such a nice work. Is written very well with consideration of all the possible analysis. This leads to a great addition of data set in the field of this study. As the results suggest a greater possibility for future analysis through experimentalist using the results suggested in this Manuscript. I found all the analysis relevant and results were interpreted very nicely. So I found the manuscript in acceptable state.

Many thanks for your kind words. We are so happy that our work admired you.

**Reviewer #4:**

Many thanks for your recommendations to make our work more valuable and scientific enough to be published. We tried to improve our work by adding your modifications and suggestions for each of the points you mentioned, taking into account everything you recommended.

1. Section 2.2-Sample Collection: Please specify the type/kind of vacutainer used, in text.

Thanks for this comment. We have specified the type of these tubes by adding the word “plain” to it.

2. Section 4- Discussion: Page 2, Paragraph 1.. "Antibiotic administered..... acid [54]."
   Please review the statement for accuracy and rephrase if required. Does the infection cause the reduction in gut microbiome diversity or the antibiotics? Further, please change nsp15 endonuclease to nsp15 endoribonuclease.

Thanks for this comment. We have modified the text so that it is now clearer. The antibiotic used in treatments causes reduction in primary bile acid metabolizing bacteria (Ref 51) and SARS-CoV-2 infection itself also reduces microbiome diversity (Ref 52 and 53).

3. The manuscript requires an overall editing in terms of consistency and grammar for tenses. Also for sub/super-scripts.

After implementing the reviewer’s comments, the manuscript has gone throughout a 2^nd^ revision for words and grammars, including tenses, by the two corresponding authors of the manuscript (Dr. Mohamed Samir, lecturer in Bath University, UK and Dr. Naglaa S. Elabd, assistant professor of Tropical medicine, Faculty of Medicine, Menoufia University, Egypt), and then the revised version was thoroughly checked by all authors. Please see some edits in the edited version of the manuscript (file name: revised manuscript with track changes).
