# Supplementary material for: Metabolic profiling during COVID-19 infection in humans: Identification of potential biomarkers for occurrence, severity and outcomes using machine learning
Source: PLoS One. 2024 May 30;19(5):e0302977. doi: 10.1371/journal.pone.0302977 (PMC11139268; doi:10.1371/journal.pone.0302977)
Supplement: Attachment — Submitted filename: Review comment.docx [file pone.0302977.s024.docx]

The authors have carefully addressed al queries. I recommend that the editor should accept the publication without any additional revision.
